# Esculeogenin A, a Glycan from Tomato, Alleviates Nonalcoholic Fatty Liver Disease in Rats through Hypolipidemic, Antioxidant, and Anti-Inflammatory Effects

**DOI:** 10.3390/nu15224755

**Published:** 2023-11-11

**Authors:** Jwharah M. Al Jadani, Nawal A. Albadr, Ghedeir M. Alshammari, Soheir A. Almasri, Farah Fayez Alfayez, Mohammed Abdo Yahya

**Affiliations:** 1 Department of Food Science and Nutrition, College of Food and Agricultural Sciences, King Saud University, Riyadh 11451, Saudi Arabia; 442203510@student.ksu.edu.sa (J.M.A.J.); aghedeir@ksu.edu.sa (G.M.A.); saalmasry@ksu.edu.sa (S.A.A.); mabdo@ksu.edu.sa (M.A.Y.); 2 Department of Medicine and Surgery, College of Medicine, King Saud University, Riyadh 11451, Saudi Arabia; farahfayezm@gmail.com

**Keywords:** esculeogenin A, nonalcoholic fatty liver disease, steatosis, obesity, high-fat diet, oxidative stress, inflammation

## Abstract

This study examined the preventative effects of esculeogenin A (ESGA), a newly discovered glycan from tomato, on liver damage and hepatic steatosis in high-fat-diet (HFD)-fed male rats. The animals were divided into six groups (each of eight rats): a control group fed a normal diet, control + ESGA (200 mg/kg), HFD, and HFD + ESAG in 3 doses (50, 100, and 200 mg/kg). Feeding and treatments were conducted for 12 weeks. Treatment with ESGA did not affect gains in the body or fat weight nor increases in fasting glucose, insulin, and HOMA-IR or serum levels of free fatty acids (FFAs), tumor-necrosis factor-α, and interleukin-6 (IL-6). On the contrary, it significantly reduced the serum levels of gamma-glutamyl transpeptidase (GGT), aspartate aminotransferase (AST), alanine aminotransferase (ALT), total triglycerides (TGs), cholesterol (CHOL), and low-density lipoprotein cholesterol (LDL-c) in the HFD-fed rats. In addition, it improved the liver structure, attenuating the increase in fat vacuoles; reduced levels of TGs and CHOL, and the mRNA levels of SREBP1 and acetyl CoA carboxylase (ACC); and upregulated the mRNA levels of proliferator-activated receptor α (PPARα) and carnitine palmitoyltransferase I (CPT I) in HFD-fed rats. These effects were concomitant with increases in the mRNA, cytoplasmic, and nuclear levels of nuclear factor erythroid 2-related factor 2 (Nrf2), glutathione (GSH), superoxide dismutase (SOD), catalase (CAT), and heme oxygenase-1 (HO); a reduction in the nuclear activity of nuclear factor-kappa beta (NF-κB); and inhibition of the activity of nuclear factor kappa B kinase subunit beta (IKKβ). All of these effects were dose-dependent effects in which a normal liver structure and normal levels of all measured parameters were seen in HFD + ESGA (200 mg/kg)-treated rats. In conclusion, ESGA prevents NAFLD in HFD-fed rats by attenuating hyperlipidemia, hepatic steatosis, oxidative stress, and inflammation by acting locally on Nrf2, NF-κB, SREBP1, and PPARα transcription factors.

## 1. Introduction

Obesity is a metabolic disorder that affects both genders and is explained by an increased calorie intake that is concomitant with reduced energy expenditure. In the last few decades, the prevalence of obesity has rapidly increased worldwide due to increasing Westernization [1]. According to recent reports by the WHO, the current prevalence of obesity in the Kingdom of Saudi Arabia has reached a pandemic-level rate of 33.7% and is expected to rise further in the coming decades [2,3,4]. However, obesity is not only associated with socioeconomic and physiological issues but is a major cause of cardiovascular and metabolic disorders, such as diabetes mellitus (DM) [5].

Nonalcoholic fatty liver disease (NAFLD) is a major clinical syndrome that affects the liver due to the increased accumulation of lipids and is characterized by a wide spectrum of liver diseases ranging from simple steatosis to nonalcoholic hepatitis (NASH), fibrosis, and liver carcinoma [6]. Studies of the etiology of NAFLD have linked this disorder to hepatocellular lipodystrophy and identified it as metabolic-associated fatty liver disease (MAFLD), which is linked to insulin resistance (IR) and metabolic syndrome (MetS) [7,8]. Indeed, the prevalence of obesity among individuals with NAFLD and NASH was found to be 51% and 81%, respectively [9]. Similarly, the prevalence of NAFLD in Americans was shown to be 67% greater in individuals with MetS [10]. In addition, the prevalence of NAFLD among patients with DM was about 60%, which increased to 77.8% in obese patients [10].

Although several clinical, preclinical, and experimental studies have been conducted, the etiology of NAFLD is not completely understood yet, and several theories and hypotheses have been generated. To date, the adipose tissue expandability hypothesis, as well as the two-hit hypothesis, remain the most acceptable methods to explain the pathogenesis of NAFLD [6,8,11]. However, increased adipose tissue mass and subsequent peripheral IR remain the driving forces for the development of NAFLD [6]. Within this review, when the capacity of the adipose tissue to store lipids is reached, the lipids are mobilized and stored in other ectopic tissues, such as the muscle and liver, which induces IR lipotoxicity and hyperglycemia [6,12]. In addition, IR and hyperglycemia further stimulate de novo hepatic lipogenesis by upregulating the activity of lipogenic transcription factors, such as the sterol regulatory element-binding protein 1 (SREBP-1) and carbohydrate responsive element-binding protein (ChREBP) [13,14]. On the other hand, a second hit of cellular and molecular changes due to increased hepatic oxidative stress and inflammation facilitates the development of NASH by promoting hepatocyte injury, lipid peroxidation, apoptosis, and fibrosis [12,14]. Within this context, metabolic inflammation due to increased inflammatory cytokines and adipokines being released from the inflamed adipose tissue, as well as the increased production of reactive oxygen species (ROS) due to an impaired oxidative phosphorylation process, mitochondria damage, and endoplasmic reticulum (ER) stress in the liver in response to lipotoxicity, is a major contributor to the hepatic-damaging effect seen in obese subjects or animals with NAFLD [6,14].

The nuclear factor (erythroid-derived 2)-like 2 (NRF-2) is the major transcription factor that contradicts cell death by increasing the expression of antioxidant enzymes and fighting oxidative stress [15]. Within the cell, Nrf2 is tightly bound with a cytoplasmic protein known as Kelch-like ECH-associating protein-1 (keap1) [16,17]. In the absence of oxidative stress, keap1 degrades Nrf2 through the ubiquitin-proteasome pathway [15]. However, in the presence of ROS, keap1 is phosphorylated and inhibited. This stimulates the nuclear translocation of Nrf2 to the nucleus to initiate the transcription process by binding to the antioxidant response elements (ARE). Major targets include superoxide dismutase (SOD), Heme oxygenase-1 (HO-1), catalase, and glutathione (GSH) thioredoxin-(TRX)-dependent enzymes [15]. In addition, Nrf2 can suppress inflammation and the production of diverse inflammatory cytokines such as tumor necrosis factor-alpha (TNF-α) and interleukine-6 (IL) by boosting the antioxidant expression of the inhibiting inflammatory transcription factor, the nuclear factor-kappa beta (NF-κB) [18]. Reduced activities of Nrf2 that were concomitant with increased oxidative stress, the decreased expression of antioxidants, lipid peroxidation, inflammation, and high activities and levels of NF-κB, IL-6, and TNF-α were observed in the livers of NAFLD animals. Currently, the lower activity of Nrf2 is listed as a major mechanism underlying NAFLD, and promoting the activation of Nrf2 is suggested to be a potent therapeutic and preventative strategy to alleviate the disease [15].

Currently, there is no definite therapy to treat or reverse NAFLD. However, weight loss via the practice of regular exercise, management of IR with the use of insulin sensitizers (e.g., thiazolidinediones (TZDs)), and statin use are the most common clinical strategies to control the progression of the disease [19,20]. Dietary antioxidants and anti-inflammatory drugs or flavonoids have been extensively studied and shown to have promising therapeutic and preventative potential [18,21,22,23]. Tomatoes (*Solanum lycopersicum*) are a major component of a healthy diet and can be consumed as whole fruits, juice, soup, and bastes [24]. In previous decades, several health benefits of tomatoes have been reported regarding their effects on cancer, DM, hypertension, infertility, and skin, renal, hepatic, and cardiovascular disorders [24]. These effects have been attributed to the hypolipidemic, antioxidant, and anti-inflammatory potential of tomatoes due to the presence of diverse phenolic compounds and flavonoids, such as lycopene, vitamins (i.e., C, E, and B12), ferulic acid, hydroxycinnamic acid, homovanillic acid, α-tomatine, tomtidine, caffeic acid, resveratrol, kaempferol, quercetin, delphinidin, phytofluene, and nucleoside [24,25]. Among these, esculeoside A, tomatine, and glycoalkaloid saponins, have received significant attention [26]. Studies have shown that esculeoside A is mainly available in mini tomatoes, whereas tomatine is more dominant in green tomatoes [27]. In ripe fruits, tomatine is oxidized at C-23 and C-27 to produce esculeoside A. However, upon ingestion, esculeoside A is also degraded into another derivative known as esculeogenin A (ESGA) [28,29].

The beneficial health effects of esculeoside A have been poorly investigated, and few studies have been conducted regarding this component. Treatment with esculeoside A has been shown to produce potent hypoglycemic activity in diabetic rats by suppressing hepatic glucose synthesis and activating peripheral and AMPK-dependent insulin signaling [26]. Additionally, esculeoside A inhibited hyaluronidase activity in vitro and showed potent antiallergenic properties in an experimental dermatitis mouse model [30]. In addition, the oral administration of esculeoside A or ESGA to apoE-deficient mice significantly lowered the serum levels of triglycerides (CHOL), cholesterol (TGs), and low-density lipoprotein cholesterol (LDL-c), and reduced foam cell formation in atherogenic lesions via hepatic acyl-CoA: cholesterol acyltransferase (ACAT) [27,28]. Very recently, esculeoside A and ESGA were shown to suppress CD4+ T lymphocyte activation by modulating the differentiation of T cells in cultured macrophages.

As a newly discovered compound, the effect of ESGA against DM and NAFLD has never been tested before. In this study, we sought to examine whether treatment with ESGA alleviates hepatic steatosis and NASH in rats fed a chronic high-fat diet (HFD). In addition, we examined some mechanisms of protection, including its effects on peripheral glucose clearance and IR as well as hepatic de novo lipogenesis and antioxidant and anti-inflammatory pathways by targeting critical transcription factors.

## 2. Materials and Methods

All animal protocols conducted in this study were approved by the Research Ethics Committee at KSU, following the US National Institutes of Health (NIH publication No. 85-23, revised 1996) (IRB: KSU-SE-22-85, approval date: 20 December 2022). Forty rats were used in this study. All rats were of the Wistar strain and were male. They were approximately 10–11 weeks old and weighed about 165 ± 15 g. The rats were supplied by the Experimental Animal Care Center at King Saud University, KSA. Throughout the whole study period, the rats were housed in a separate room in plastic cages (8 rats/cage) under ambient temperature (23 ± 1 °C) and a 12 h dark/light cycle.

### 2.1. Diets

Both control (standard) (Cat. # D12450J, Research Diets, New Brunswick, NJ, USA) (3.8 kcal/gm; 10 kcal% fat, 20 kcal% protein, 70 kcal% carbohydrates) and HFD (Cat. # D12492, Research Diets, NJ, USA) (5.2 kcal/gm; 60 kcal% fat, 20 kcal% protein, 20 kcal% carbohydrates) groups were used in this study. This HFD was previously used to induce NAFLD in mice [31,32] and rats [33,34] after 12–16 weeks of chronic administration. The compositions of both diets can be found online on the supplier’s webpage.

### 2.2. Preparation of the ESGA

The isolation of ESGA was prepared from fresh, ripe, red cherry tomato fruits at the College of Pharmacy of KSA, as described by others. In brief, the tomato fruits were bought fresh from a local farm in the Kingdom of Saudi Arabia during the cultivation period. These fruits were cut, washed, cut again into smaller pieces, and then smashed, filtered, and centrifuged at 500× *g* for 10 min. First, we isolated the esculeoside A fraction using column chromatography. For this, the freshly isolated supernatants were passed through a Diaion HP20 (Cat. # 13616, Aldrich, St Louis, MO, USA) and then eluted using 40% and 60% methanol. The identified esculeoside A was hydrolyzed using 2N HCL and extracted using ethyl acetate. The organic layer was removed under vacuum, and the resultant pellet was purified using silica gel chromatography with chloroform/methanol/H2O (9:10:1 *v*/*v*). The structure of the final product was checked at the College of Pharmacy and was similar to the structure published by others and identified as ESGA. This was kept at −4 °C and used for the experimental procedure.

### 2.3. Experimental Design

Six groups were created in this study, each containing 8 rats. The isolated ESGA was freshly dissolved in 5% carboxymethyl cellulose (CMC). The groups were (1) a control group, which was fed a normal, standard diet and orally administered 5% CMC as a vehicle for a total period of 12 weeks; (2) ESGA-treated rats (200 mg/kg), which were fed a normal, standard diet and co-treated orally with the highest dose of ESGA at a final dose of 200 mg/kg for 12 weeks; (3) the HFD model group, which was fed HFD and co-treated orally with the 5% CMC solution for 12 weeks; (4) the HFD + ESGA (50 mg/kg)-treated group, which was fed HFD and co-treated orally with ESGA at a final dose 50 mg/kg; (5) the HFD + ESGA (100 mg/kg)-treated group, which was fed HFD and co-treated orally with ESGA at a final dose 100 mg/kg; and (6) the HFD + ESGA (200 mg/kg)-treated group, which was fed HFD and co-treated orally with ESGA at a final dose of 200 mg/kg. Body weight and food intake were monitored every week. Previous studies have shown that treatment with ESGA induces a dose–response atherogenic protection and the attenuation of serum lipids in mice fed an HFD [28]. The safety of ESGA was tested in our laboratories, and no signs of toxicity were shown up to a dose of 5000 mg/kg. The oral treatment with ESGA was administered via gavage using a stainless-steel feeding cannula.

### 2.4. Analysis of Blood Glucose and Insulin Levels and Assessment of HOMA-IR

At the end of all treatments, the rats fasted overnight, and EDTA blood samples were withdrawn directly via cardiac puncture (1 mL). All samples were centrifuged at 500× *g* for 10 min to collect plasma. Levels of plasma glucose were measured at all tested intervals using a glucose assay kit (Cat. # 10009582, Cayman Chemicals, Ann Arbor, MI, USA). The plasma glucose levels were measured at 0.0 min using an insulin ELISA kit (cat # ERINS, ThermoFisher, Waltham, MA, USA). The Homeostasis Model Assessment of Insulin Resistance (HOMA-IR) was calculated from the basal glucose and insulin levels, as described previously in our laboratory [35]. All analyses were performed in duplicate for *n* = 8 samples/group as per the recommendations of the manufacturer.

### 2.5. Liver Function Tests (LFTs)

Two days after the intraperitoneal glucose tolerance test (IPGTT), the rats were fasted again and then anesthetized using ketamine (65 mg/kg). Overnight blood samples (1 mL) were withdrawn via cardiac puncture into gel-containing tubes to collect serum (1200× *g*/10 min/room temperature). All serum samples were preserved at −20 °C until further use. The levels of major liver function components, including gamma-glutamyl transpeptidase (GGT), aspartate aminotransferase (AST), and alanine aminotransferase (ALT), in these serum samples, were measured using special commercial ELISA kits for rats, including a GGT, AST, and ALT determination ELISA kit for rats (Cat. # MBS269614; Cat. # MBS264975; Cat. # MBS264975; MyBioSorces, San Diego, CA, USA, respectively). The analyses were performed in duplicate for *n* = 8 samples/group as described in each kit.

### 2.6. Markers of Adipose Tissue Function and Inflammation

Serum levels of leptin were measured using a special rat leptin ELISA kit (Cat. # ab100773, Abcam, Cambridge, UK). Serum adiponectin levels in all collected samples were analyzed using a rat adiponectin ELISA kit (Cat. # ab239421, Abcam, Cambridge, UK). The levels of interleukin-6 (IL-6) and tumor necrosis factor-alpha (TNF-α) in the serum were measured using special rat IL-6 and TNF-α ELISA kits (Cat. # MBS269892 and Cat. # MBS9501941, MyBioSorces, San Diego, CA, USA, respectively). All measurements were analyzed in duplicate with 8 samples per group following the instructions of each kit.

### 2.7. Tissue and Fat Pad Collection

The cervical dislocation was used for euthanasia. Livers and adipose tissue fat (epididymal, retroperitoneal, and subcutaneous) pads were collected. All collected tissues were weighted, cut into smaller sections, and kept at −80 °C until use. Some parts of the liver were preserved in 10% buffered formalin for histological evaluation.

### 2.8. Lipid Analysis in the Serum and Liver

The intrahepatic lipids were extracted from the livers using the chloroform/methanol method established by Folch et al. [36] and described by us and others [32,37]. In brief, liver samples (100 mg) were homogenized with 0.5 mL PBS, and total lipids were extracted via the addition of 1.5 mL of a 2:1 (*v*/*v*) chloroform:methanol mixture. All samples were then centrifuged at 2500× *g* for 15 min at 4 °C, followed by the separation of the lower layer containing the lipids. Under vacuum, the solvent was evaporated, and the collected pellet containing the lipids was dissolved in a 1 mL ethanol solution containing 1% Triton X. Triglycerides (TGs) in the serum and lipid extract were measured using a TG assay kit (Cat # 10010303, Cayman Chemicals, Ann Arbor, MI, USA). Serum and liver levels of low-density lipoprotein cholesterol (LDL-c) were assessed using an LDL-c colorimetric kit (Cat. # 79980, Crystal chem, Elk Grove Village, IL, USA). Serum levels of total cholesterol (CHOL) and free fatty acids (FFAs) were measured using colorimetric assay kits (Cat. # BA0064 and Cat. # BA0101, AssayGenie, Dublin, Ireland). All measurements were performed for a duplicate of 8 samples per group and as per the manufacturer’s instructions.

### 2.9. Analysis of Markers of Oxidative Stress and Inflammation in Liver Homogenates

Parts of the liver tissues (75 mg) were homogenized in cold PBS (pH = 7.4), and supernatants (homogenates) were collected (12,000× *g*/20 min/4 °C). The levels of TNF-α and IL-6 were measured using the same ELISA kits used to determine their levels in the serum. The concentration of malondialdehyde (MDA) was determined using a commercial MDA analysis kit (Cat. # K739-100, Biovision, Waltham, MA, USA). The total levels of major enzymatic antioxidants, including heme oxygenase-1 (HO-1), were measured using a rat Ho-1 ELISA kit (Cat # ADI-EKS-810A; ENZO, Farmingdale, NY, USA); the total levels of catalase and SOD1 were analyzed using specialized CAT and SOD-1 ELISA kits (Cat. No E0869Ra and Cat. No. E1444Ra, Bioassay Technology Laboratory, Shanghai, China, respectively). Total GSH concentration was analyzed using a rat-specific GSH ELISA kit (Cat. # RTEB1811, AssayGenie, Dublin, Ireland). All procedures were conducted for *n* = 8 liver samples/group (duplicate) and as instructed by the recommendations for each kit.

### 2.10. Assessment of Nuclear Activities of Nrf2 and NF-κB

To evaluate the nuclear translocation of Nrf2 and NF-κB, we calculated the ratio of their nuclear/cytoplasmic levels. In brief, the nuclear and cytoplasmic fractions of the liver samples were prepared using the Minut Cytosolic and Nuclear Extraction Kit for Frozen/Fresh Tissues (Cat. # NT-032; Invent Biotechnologies, Plymouth, MN, USA). The assessment of the cytoplasmic and nuclear levels of Nrf2 and NF-κB was conducted using ELISA kits (Cat. # NBP3-08161, Novus biologicals, Centennial, CO, USA and Cat. # CSB-E13148r, CUSABIO, Houston, TX, USA, respectively).

### 2.11. Real-Time Polymerase Chain Reaction (q-pCR)

Real-time PCR was performed, as previously described, in our laboratory [35]. Total RNA was extracted from frozen samples using the TRIZOL reagent (ThermoFisher Scientific). RNA samples were treated with DNase, and the purity of DNA was determined by reading the absorbance at 260/280 nm using a nanodrop. A RevertAid First Strand cDNA Synthesis Kit (Cat. # K1621, ThermoFisher Scientific) was used to synthesize the first-strand cDNA. The amplification reactions were performed using the Ssofast Evergreen Supermix kit (Cat. # 172-5200, BioRad, Hercules, CA, USA) with the Step One Plus Real-Time PCR system (Applied Biosystems, Carlsbad, CA, USA). The sequences of the primers used in this study are shown in Table 1. The mRNA expression levels of all selected genes were normalized against actin as an internal control.

### 2.12. Liver Histology

All liver samples were preserved in 10% buffered formalin for 10 min. The next day, all liver samples were treated with xylene and descending concentrations of ethanol. All samples were then embedded and sliced into 3–5 µM sections. Then, all samples were routinely stained with Harris hematoxylin/glacial acetic acid solution and washed with HCL in ethanol (1:400 *v*/*v*). They were then stained with eosin, dried, and covered with a mounting media and coverslips. All slides were examined and photographed using a light microscope.

### 2.13. Statistical Analysis

The statistical analysis for all measured parameters was conducted using the GraphPad Prism statistical software package (version 8) and a one-way ANOVA test. Normality was tested using the Kolmogorov–Smirnov test, and Tukey’s test was used as a post hoc test. Data are presented as the mean ± SD. The value of significance was set at *p* < 0.05.

## 3. Results

### 3.1. ESGA Does Not Affect Body and Fat Weight, Hyperglycemia, Hyperinsulinemia, and IR, but Significantly Reduces Liver Weights in HFD-Fed Rats

The final body weight, liver weight, liver/body weight percentage, the final weight of all fat pads (subcutaneous, epididymal, and peritoneal), the serum levels of FFAs, leptin, TNF-α, and IL-6 (Table 2), and fasting glucose and insulin levels, as well as levels of HOMA-IR (Table 2), showed significant increases (*p* < 0.001) in the livers of HFD-fed rats as compared to the control and ESGA-treated rats. Except for liver weights, the levels of these markers were not significantly different (*p* > 0.5) when HFD + ESAG-treated rats (all doses: 50, 100, and 200 mg/kg) were compared with each other or with the control or HFD-fed rats (Table 2). Additionally, treatment of the control rats with ESAG (200 mg/kg) did not significantly affect the levels of these markers. On the other hand, liver weights and liver-to-final body weight percentages were progressively and significantly reduced with all ESGA treatments compared to HFD-fed rats (*p* < 0.05) (Table 1). The liver weights remained significantly higher in HFD + ESGA (50 and 100 mg/kg)-fed rats compared to control rats but were not significantly different when HFD + ESGA (200 mg/kg)-fed rats were compared to control rats *(p* > 0.05). Also, the liver-to-final body weight percentages were not significantly different between the control and HFD + ESGA (50 mg/kg)-fed rats but were significantly lower in HFD + ESGA (100 and 200 mg/kg)-fed rats compared to control rats in the induced NASH animal model (Table 1).

### 3.2. ESGA Attenuates the Increase in Liver Function Test Enzymes in the Serum of HFD-Fed Rats

There were no significant differences in the serum levels of ALT, AST, and GTT between the ESGA-treated rats (200 mg/kg) and control rats (Table 3). The HFD-fed rats showed significant increments in their levels of ALT, AST, and GTT, compared with the control and ESGA-treated rats (*p* < 0.001) (Table 3). The levels of these enzymes decreased progressively in a dose-dependent manner in the serum of HFD-fed rats that were co-administered with ESGA (50, 100, and 200 mg/kg) (*p* < 0.05) (Table 3). The serum levels of these enzymes were not significantly different between the HFD + ESGA-treated group (50, 100, and 200 mg/kg) and the HFD model group (Table 3) (*p* > 0.05).

### 3.3. ESGA Represses Serum and Hepatic Lipids but Not Stool Lipids in Control and HFD-Fed Rats

Serum levels of FFAs, total TGs, total CHOL, and LDL-c, as well as the hepatic and stool levels of TGs and CHOL, were significantly higher in the HFD-fed group of rats compared to the control and ESGA-treated rats (*p* < 0.001) (Table 4). Serum and hepatic levels of all of these lipids were significantly reversed in a progressive and dose-dependent manner in HFD + ESAG-treated rats that were administered ESGA at increasing doses (50, 100, and 200 mg/kg) (Table 4). Only in the latter group did the serum and hepatic levels of all these lipids show no significant variation (*p* > 0.5) compared with those of the control rats (Table 4). In addition, no significant variations (*p* > 0.5) in the stool TGs and CHOL levels were seen when ESGA-treated rats were compared with control rats or when HFD + ESGA-treated rats (50, 100, and 200 mg/kg) were compared with the HFD group (Table 3). Of note, the serum and hepatic levels of FFAs, total CHOL, and total TGs, as well as serum levels of LDL-c, were significantly depleted (*p* < 0.01) in ESGA-treated control rats (200 mg/kg) compared with the control group in the study (Table 4).

### 3.4. ESGA Stimulates the Nrf2/Antioxidant Axis in the Livers of Control and HFD-Fed Rats

**Figure 1:**
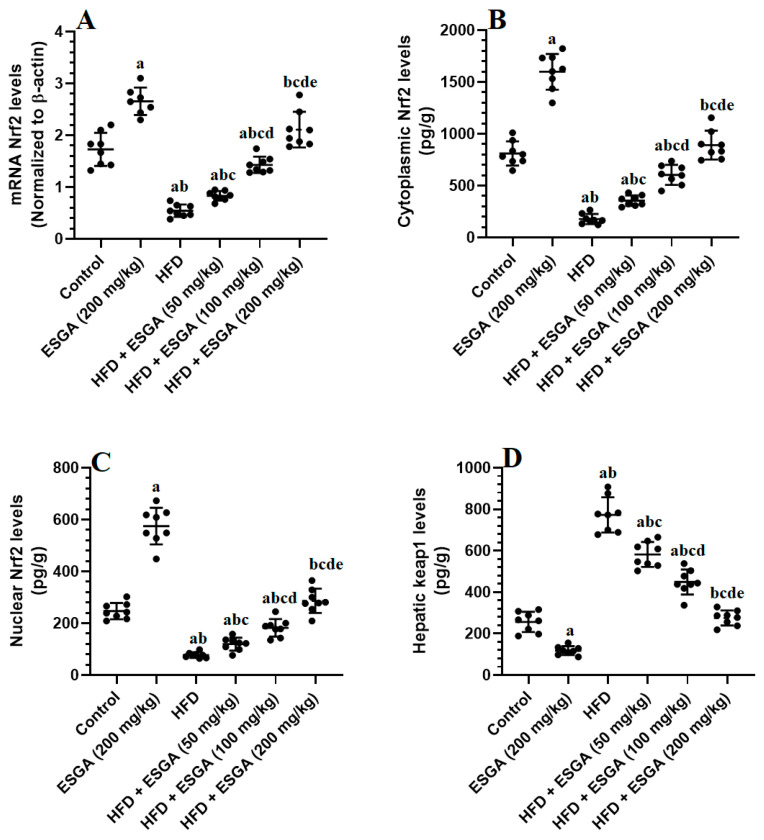
mRNA of Nrf2 (**A**), total cytoplasmic levels of Nrf2 (**B**), nuclear level of Nrf2 (**C**), and total level of keap1 (**D**) in the liver homogenates of all experimental groups. Data were analyzed via one-way ANOVA and Tukey’s post hoc test and are presented as the mean ± standard deviation (SD) for *n* = 8 samples/group. The level of significance was considered to be *p* < 0.05. ^a^ Significantly different from the control rats fed the standard diet. ^b^ Significantly different from the esculeogenin A (ESGA)-treated rats. ^c^ Significantly different from the high-fat diet (HFD)-fed rats. ^d^ Significantly different from the HFD + ESGA (50 mg/kg)-fed rats. ^e^ Significantly different from the HFD + ESGA (100 mg/kg)-fed rats.

**Figure 2:**
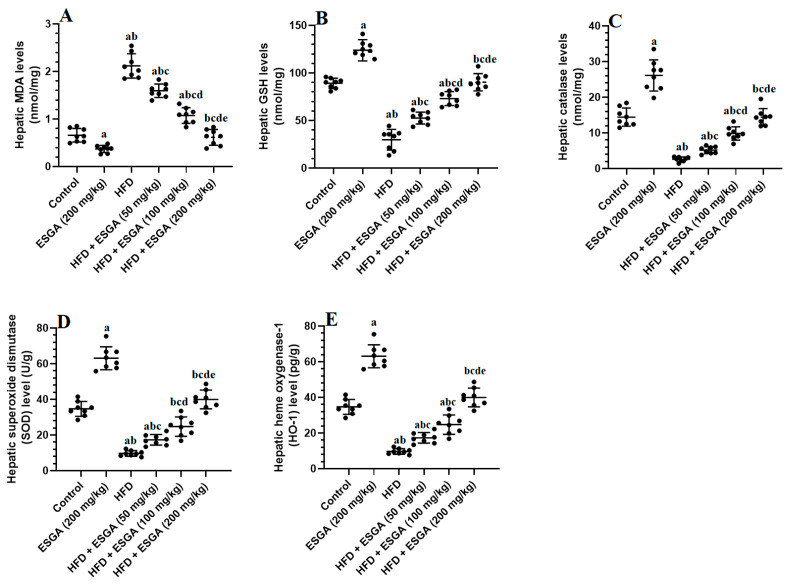
Levels of malondialdehyde (MDA) (**A**), a marker of lipid peroxides, and the levels of some antioxidants, including glutathione (GSH) (**B**), catalase (**C**), superoxide dismutase (SOD) (**D**), and heme oxygenase (HO-1) (**E**), in the liver homogenates of all experimental groups. Data were analyzed via one-way ANOVA and Tukey’s post hoc test and are presented as the mean ± standard deviation (SD) for *n* = 8 samples/group. The level of significance was considered to be *p* < 0.05. ^a^ Significantly different from the control rats fed the standard diet. ^b^ Significantly different from the esculeogenin A (ESGA)-treated rats. ^c^ Significantly different from the high-fat diet (HFD)-fed rats. ^d^ Significantly different from the HFD + ESGA (50 mg/kg)-fed rats. ^e^ Significantly different from the HFD + ESGA (100 mg/kg)-fed rats.

There were significant reductions (*p* < 0.01) in the mRNA, cytoplasmic, and nuclear levels of Nrf2 and levels of GSH, SOD, catalase, and HO-1, in parallel with significant increments (*p* < 0.001) in the cytoplasmic levels of keap1 and MDA in the livers of HFD-fed rats compared with the control and ESGA-treated groups (Figure 1A–D and Figure 2A–E). The levels of MDA and keap1 were significantly reduced (*p* < 0.05), while the levels of all other parameters mentioned above were significantly increased (*p* < 0.05) in a dose-dependent fashion in the livers of HFD-treated rats that were co-administered ESGA (50, 100, and 200 mg/kg) (Figure 1A–D and Figure 2A–E). Similar reductions in the levels of MDA and cytoplasmic keap1 were concomitant with increases in the mRNA, cytoplasmic, and nuclear levels of Nrf2 and the levels of antioxidant enzymes observed in the livers of ESGA-treated rats (200 mg/kg) compared with control rats (Figure 1A–D). However, the levels of all chemical endpoints were not significantly different (*p* > 0.05) when a comparison was made between the standard control group and the HFD + ESGA group (200 mg/kg).

### 3.5. ESGA Suppresses the IKK/NF-κB p65/Inflammatory Pathway of Nrf2/Antioxidant in the Livers of Control and HFD-Fed Rats

**Figure 3:**
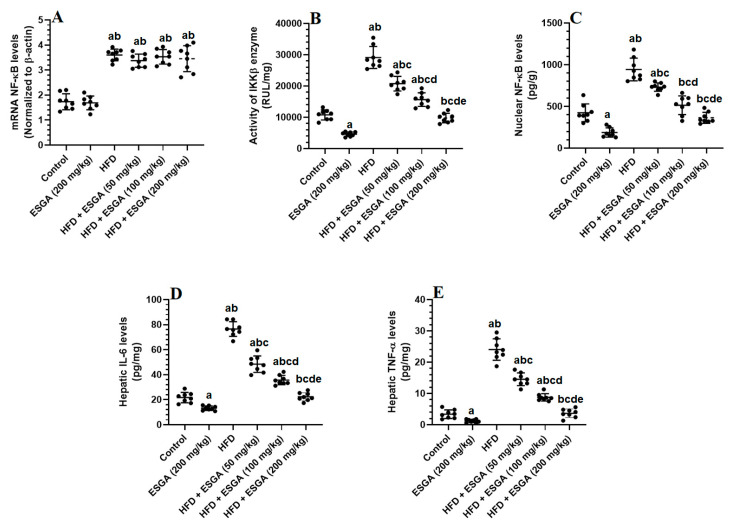
mRNA levels of NF-κB (**A**), the activity of the inhibitor of nuclear factor kappa B kinase subunit beta (IKKβ) (**B**), the nuclear level of NF-κB (**C**), and the levels of interleukin-6 (**D**) and tumor necrosis factor-alpha (TNF-α) (**E**) in the livers of all experimental groups. Data were analyzed via one-way ANOVA and Tukey’s post hoc test and are presented as the mean ± standard deviation (SD) for *n* = 8 samples/group. The level of significance was considered to be *p* < 0.05. ^a^ Significantly different from the control rats fed the standard diet. ^b^ Significantly different from the esculeogenin A (ESGA)-treated rats. ^c^ Significantly different from the high-fat diet (HFD)-fed rats. ^d^ Significantly different from the HFD + ESGA (50 mg/kg)-fed rats. ^e^ Significantly different from the HFD + ESGA (100 mg/kg)-fed rats.

The hepatic mRNA of NF-κB, as well as the total activity of IKKβ, the nuclear levels of NF-κB p65, and the levels of TNF-α and IL-6, were significantly higher (*p* < 0.001) in the livers of HFD model rats compared to those in the control and ESGA (200 mg/kg)-treated rats (Figure 3A–E). The hepatic mRNA levels of NF-κB were not significantly different (*p* > 0.05) when the control rats were compared with the ESGA (200 mg/kg)-treated rats or when the HFD-fed rats were compared with the HFD + ESGA (50, 100, and 200 mg/kg)-fed rats (Figure 3A–E). On the contrary, there were significant and progressive declines (*p* < 0.05) in the activity of IKKβ, the nuclear level of NF-κB p65, and the levels of IL-6 and TNF-α in the livers of HFD-fed rats that received various doses of ESGA (50, 100, and 200 mg/kg) when compared with the HFD model group (Figure 3A–E). The maximum reductions in the levels/activities of these markers were seen in the livers of HFD + ESGA (200 mg/kg)-fed rats, but these were not significantly different when compared with control rats fed the standard diet (Figure 3A–E).

### 3.6. ESGA Suppresses the SREBP1/ACC Axis and Stimulates the PPARα/CPT-1 Axis in the Livers of the Control and HFD-Fed Rats

**Figure 4:**
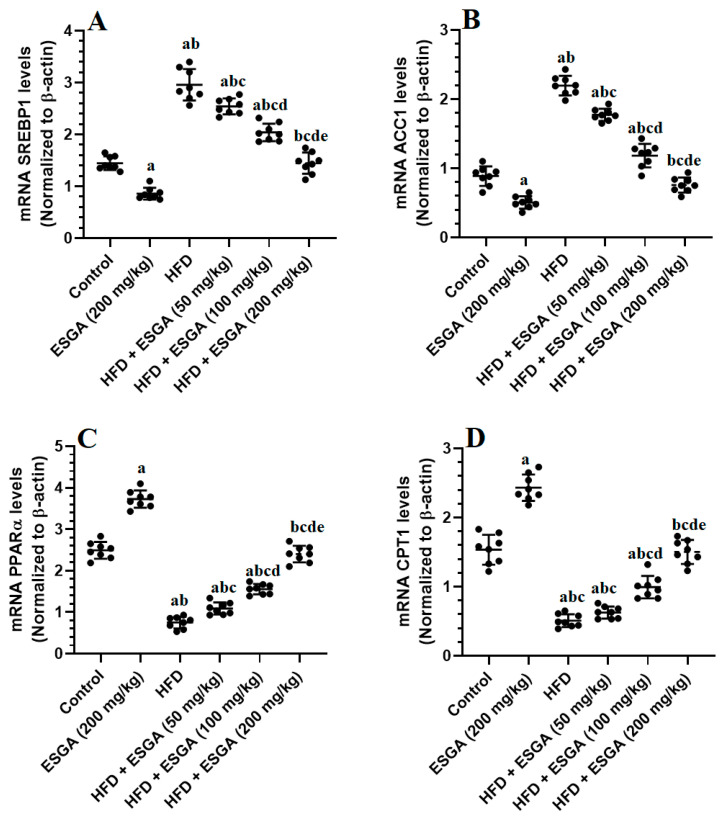
mRNA levels of SREBP1 (**A**), ACC-1 (**B**), PPARα (**C**), and CPT-1 (**D**) in the livers of all experimental groups. Data were analyzed via one-way ANOVA and Tukey’s post hoc test and are presented as the mean ± standard deviation (SD) for *n* = 8 samples/group. The level of significance was considered to be *p* < 0.05. ^a^ Significantly different from the control rats fed the standard diet. ^b^ Significantly different from the esculeogenin A (ESGA)-treated rats. ^c^ Significantly different from the high-fat diet (HFD)-fed rats. ^d^ Significantly different from the HFD + ESGA (50 mg/kg)-fed rats. ^e^ Significantly different from the HFD + ESGA (100 mg/kg)-fed rats.

The mRNA levels of SREBP1 and ACC were significantly increased, while the mRNA levels of PPARα and CPT-1 were significantly reduced in the livers of HFD model rats compared to control rats (Figure 4A–D). An opposing image of the expression of all of these markers was seen in the livers of ESGA (200 mg/kg)-treated rats compared to the control group and in the livers of HFD + ESGA (50, 100, and 200 mg/kg)-fed rats compared to HFD-fed rats. The effect was maximal in the livers of HFD-fed rats that were co-treated with the highest dose of ESGA (200 mg/kg), compared to all other treated rats (i.e., 50 and 100 mg/kg), which were not significantly different from the control rats (Figure 4A–D).

### 3.7. ESGA Improves Histological Features and Reduces Ballooning in the Hepatocytes of HFD-Fed Rats

**Figure 5:**
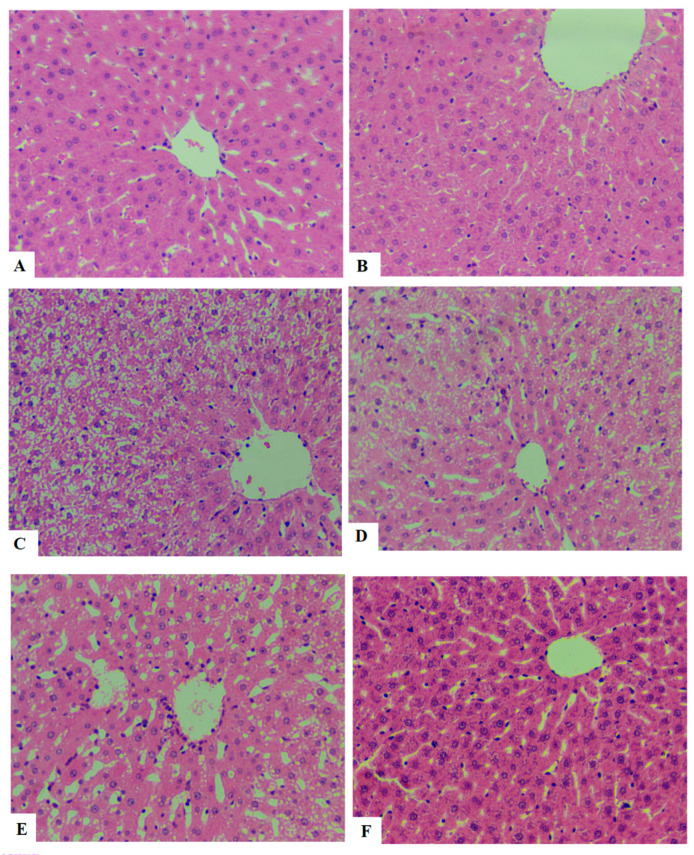
Morphological images for the livers of all groups of rats after staining with hematoxylin and eosin (magnification = 200×). (**A**,**B**) are taken from the control rats and esculeogenin A (ESGA)-treated rats, respectively, and show normal hepatocytes with rounded nuclei. (**C**) is from HFD-fed rats and shows a massive increase in the number of cytoplasmic fat vacuoles (ballooning) in the majority of the hepatocytes (black arrows). (**D**–**F**) are taken from the HFD + esculeogenin A-treated rats (50, 100, and 200 mg/kg, respectively), and show a gradual improvement in the structure of the hepatocytes that is characterized by a reduction in the size and number of cytoplasmic fat vacuoles with almost normal hepatocytes with no definite fat vacuoles in F (200 mg/kg).

Normal hepatic features with intact hepatocytes, central veins, and sinusoids were seen in the livers of control and ESGA (200 mg/kg)-treated control rats (Figure 5A,B). Damaged hepatocytes with increased cytoplasmic lipid vacuoles were seen in the livers of HFD-fed rats (Figure 5C). A progressive improvement in the liver structure with obvious reductions in the size and number of fat vacuoles was seen in HFD-fed rats that were co-treated with increasing doses of ESGA (50, 100, and 200 mg/kg) (Figure 5D–F). Almost normal liver features were seen in HFD + ESAG (200 mg/kg)-treated rats (Figure 5F).

## 4. Discussion

**Figure 6:**
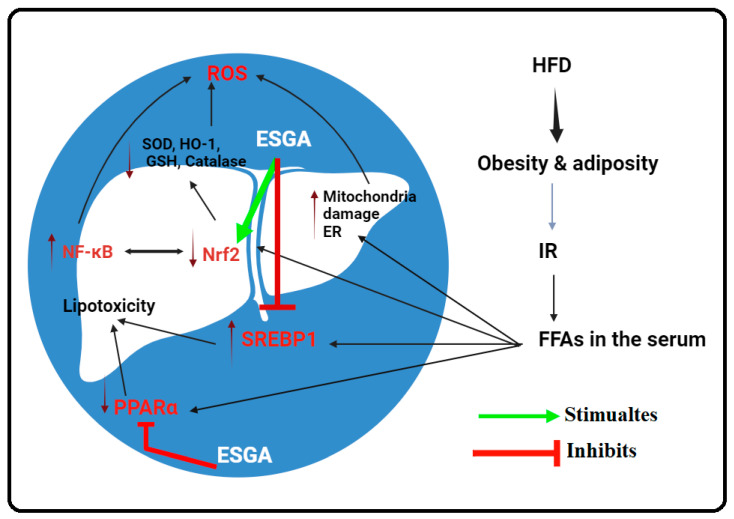
A graphical abstract demonstrating the protective mechanisms of esculeogenin A (ESGA) associated with alleviating nonalcoholic fatty liver disease. In the graph, ESGA is shown to act via three major mechanisms, namely, (1) stimulating Nrf2, (2) downregulating SREBP1, and (3) inhibiting PPARα.

Recent research advances have focused on treating and preventing chronic and metabolic disorders, given their rapid increases and negative impacts on the socioeconomic and health systems. The data from this study list ESGA as a novel natural molecule that can alleviate hepatic damage in animals with NAFLD through modulating hyperlipidemia and hepatic steatosis, oxidative stress, and inflammation. The hepatic protective effect of ESGA was found to be dose-dependent and with no effect on adiposity, IR, and hyperglycemia, and act through modulating the expression and activities of hepatic transcription factors, including SREBP1/PPARα lipogenic, Nrf2/antioxidant, and the NF-κB/inflammatory axis. A graphical abstract clarifying these effects is shown in Figure 6.

When studying NAFLD, a major concern is the correct selection of the animal model to correctly reflect similar clinical symptoms to those seen in humans [34,38]. Currently, the transgenic and nutritional animal models are the most common models used to study the pathogenesis of and effects of treatments on NAFLD in rodents [34]. This is because of their ability to induce substantial metabolic and histological similarities to those effects seen in individuals with NAFLD and NASH by promoting obesity, IR, and hyperlipidemia, increasing hepatic lipid uptake and lipogenesis, decreasing lipid exportation from the liver, and reducing FA oxidation and impairing mitochondrial oxidative phosphorylation [34,38]. Using these animal models, severe hepatic pan lobar steatosis, ballooning, inflammation, fibrosis, and necrosis are produced, resulting in increases in liver function enzymes, such as ALT, AST, and GTT, in the serum by up to 20-fold [34,38,39,40]. In addition, they increase circulatory inflammatory cytokines/adipokines (e.g., TNF-α, IL-6, C-reactive protein, and leptin) from the insulin-resistant adipose tissue and damaged liver, which are considered to be valid screening tools for monitoring the progression of the disorder [41,42]. Nonetheless, among all nutritional diets used, HFD-fed animal models that depend on the chronic feeding of a diet rich in fats (30–75 kcal), given as either a pellet or liquid, have received the greatest interest. However, the degree of hepatic steatosis and liver damage depends on several factors, such as the animal species, sex, feeding period, physical activity, and genetic background of the animal used [34,43,44]. In general, a total feeding period of 16 weeks is sufficient to induce NASH in both mice and rats [45]. In addition, when using the same HFD chronic feeding regimen, rats are more susceptible to the development of NAFLD and require a shorter treatment period to induce NASH, with more severe histological abnormalities compared to mice [38,46]. In addition, adult male Sprague Dawley rats are more susceptible to the development of hepatic steatosis, ballooning, inflammation, and fibrosis over a shorter period, compared to female Sprague Dawley rats and the Wistar rat species [34,47,48]. Some studies have shown that chronic HFD feeding does not induce hepatic steatosis or NASH in Wistar rats [47].

For these reasons, we used adult male Sprague Dawley rats in this study and followed the recommended HFD-feeding protocol for 16 weeks. In addition, we validated our HFD obesity-NAFLD animal model by reporting significant increments in the rats’ final body and fat pad weights, fasting hyperglycemia, hyperlipidemia, hyperinsulinemia), higher HOMA-IR (a marker of IR), increases in the serum levels of ALT, AST, and GTT, and via the obvious degeneration and ballooning in the hepatocyte structure. In addition, we found a significant increase in the serum level of FFAs, which corresponds to IR in these rats. In addition, in this study, HFD-fed rats also showed increased levels of serum leptin, which is normally released and correlated with adipose tissue, which explains the observed polyphagia in rats [49]. Furthermore, HFD-fed rats showed significant increases in their levels of TNF-α and IL-6, suggesting a state of chronic inflammation in the liver and adipose tissues. On the other hand, treatment with ESGA at increasing doses of 50, 100, and 200 mg/kg, failed to attenuate or reverse obesity and did not prevent increases in fat pad accumulation, glucose and insulin levels, HOMA-IR levels, or the circulatory levels of leptin in HFD-fed rats. However, they induced dose–response protective and ameliorative effects on the degree of hepatic steatosis, liver damage, and circulatory levels of ALT, AST, GTT, TNF-α, FFAs, and IL-6. These data provide initial evidence that the protective effect of ESGA is mediated by local hepatic responses rather than by attenuating obesity and peripheral IR, which are considered to be major triggers of NAFLD [50]. In general, some studies have shown the hypoglycemic effects of red and green tomatoes in animal models of type 1 and type 2 DM, mainly through improving the IR in the adipose tissue and muscles [51]. These effects have been attributed to certain ingredients, such as lycopene and esculeoside A [51]. Additionally, treatment with eculeoside A alone attenuated fasting hyperglycemia in dp/dp mice via the AMPK-induced expression of insulin receptors on peripheral tissues [26]. However, our findings also generate this result and suggest that the hypoglycemic and insulin action-improving effects of esculeoside A are not related to its intestinal conversion to ESGA but could be mediated by other metabolites.

Dyslipidemia, which is seen in obesity and metabolic syndrome, plays a significant role in the development of NAFLD and NASH and is a major risk factor for increased mortality due to cardiovascular disorders (CVDs) among these patients [52]. The clinical dyslipidemia findings in patients and animals with NAFLD are characterized by hypertriglyceridemia, increased circulatory LDL and VLDL-c levels, and low levels of HDL-c; a condition known as atherogenic dyslipidemia [53]. Hypolipidemic agents, such as statins, attenuate hepatic steatosis and reduce the risk of cardiovascular disorders [52,53,54]. In the liver, lipid synthesis is a tightly regulated mechanism that involves a delicate balance between de novo lipogenesis (DNL) and FA oxidation, controlled by numerous transcription factors [55,56]. In general, the transcription factor SREBP1 stimulates DNL by increasing the transcription of several lipogenic genes, such as fatty acid oxidase and acetyl CoA carboxylase (ACC) [57]. Of interest, the hepatic expression of SREBP1c induces hepatic steatosis, and the deletion of this factor protects HFD-fed rats [58,59]. Higher levels of SREBP1c have also been seen in HFD-fed animals [14,37,60,61]. On the other hand, PPARα stimulates FA oxidation by increasing the mitochondrial uptake of FFAs by activating the carnitine palmitoyltransferase enzymes (i.e., CPT1 and CPT-2) [62]. In addition, PPARα exerts potent antioxidant and anti-inflammatory effects in a majority of tissues, including the liver, and can suppress the generation of various inflammatory cytokines and ROS by negatively regulating NF-κB [63,64,65]. Significant reductions in the levels of PPARα and CPT1/2 have been documented in the livers of HFD-fed animals and are considered a major mechanism for the development of steatosis [66,67]. The gene-specific deletion of PPARα promotes NAFLD, whereas the expression of PPARα and the use of PPARα agonists, such as fenofibrate, prevent hepatic steatosis, inflammation, oxidative stress, and liver injury in HFD-fed animals [66,68,69].

Dyslipidemia was also evident in the serum and livers of the HFD-fed rats, which showed a typical portrayal of atherogenic dyslipidemia (i.e., high CHOL, TGs, and LDL-c levels). At the same time, their livers showed higher levels of FFAs, TGs, and CHOL, that were concomitant with a high number of fat vacuoles. Supporting the above-mentioned studies, the livers of HFD-fed animals also showed higher mRNA levels of SREBP1 and ACC1 that were associated with the reduced transcription of PPARα and ACC1 compared to their levels in control rats. These results can be explained by the hyperglycemia, hyperinsulinemia, and hyperadiponectinemia observed in these rats, as well as by the higher hepatic levels of ROS and inflammatory cytokines. Indeed, hyperinsulinemia, hyperglycemia, ROS, and inflammatory cytokines can rapidly upregulate and activate SREBP1c and cause severe hepatic steatosis, as seen in obesity, NAFLD, and the early stages of T2DM [56,70,71,72]. Opposing REBP1c, insulin, and growth hormones stimulate this condition, whereas adiponectin stimulates hepatic PPARα [73,74,75]. Therefore, the hyperinsulinemia and adiponectin resistance observed in HFD-fed animals in this study triggered the suppression of the PPARα/CPT1 axis [56]. On the other side of this study, dose–response reductions in the serum and hepatic levels were also observed in the livers of all rats fed an HFD and co-treated with ESGA and were associated with similar dose–response reversals in the hepatic expression of SREBP1, ACC, PPARα, and CPT1. Interestingly, while FFA levels remained unchanged in the serum of HFD + ESGA-treated groups at all doses, they were significantly reduced in the livers of these rats, which could be explained by the inhibitory effect of ESGA on SREBP1 activity and the stimulation of PPARα-induced FA oxidation. In addition, the reductions in the serum and hepatic levels of TGs, CHOL, and LDL-c post-treatment with ESGA were not related to the modulation of intestinal fat absorption, as higher levels of TGs and CHOL were also found in the stools of all ESGA-treated rats, which were not significantly different from each other or from those measured in the stools of HFD-fed rats. In addition, and as discussed before, this hypolipidemic effect is independent of the modulation of obesity, IR, adiponectinemia, and hyperglycemia, and seems to be an independent effect that acts mainly on the liver SREBP1 and PPARα axes and could be a direct effect or indirectly mediated by the alleviation of oxidative stress and inflammation.

On the other hand, considering the antioxidant and anti-inflammatory effects of PPARα, it could be possible that the activation of PPARα is an additional mechanism underlying the antioxidant and anti-inflammatory effects of ESAG discussed later. To our knowledge, these data are the first to show these mechanistic hypolipidemic effects of ESGA in animals or humans. However, the mechanism by which ESGA reduces serum CHOL remains unclear based on the data from this study. In previous research, some authors have shown that ESGA attenuates hypercholesterolemia and hepatic cholesterol lipid synthesis by suppressing acyl-coenzymeA (CoA): cholesterol acyl-transferase (ACAT) in apoE-deficient mice and by attenuating IR in HFD-fed rats [28]. Therefore, our study reveals more mechanisms at play and highlights a regulatory effect of this drug transcription factor on lipid synthesis and oxidation.

However, oxidative stress and inflammation are two key central mechanisms underlying the progression of NAFLD to NASH [6,8]. Higher levels of ROS and inflammatory cytokines are generated in the livers of HFD-fed animals due to the effects of peripherally released cytokines, the reduced levels of circulatory anti-inflammatory adipose tissue-derived adiponectin, hepatic lipotoxicity-induced endoplasmic reticulum (ER) stress, and impairments of the mitochondrial structure and function [8,76]. However, all cells, including the hepatocytes, are well equipped with endogenous systems to fight these pathological mechanisms [76]. NF-κB is the major cytoplasmic inflammatory transcription factor that induces chronic inflammation and generates large quantities of ROS by stimulating the transcription of several inflammatory cytokines, such as TNF-α and IL-6 [77]. In addition, NF-κB p65 participates directly in and stimulates hepatic lipid synthesis by repressing the transcription of sorcin, which stimulates the nuclear translocation and activation of the lipogenic transcription factor carbohydrate response element-binding protein (ChREBP) [78]. The nuclear activation of NF-κB is inhibited by binding to a cytoplasmic protein known as IκB [79]. However, under stimulation by ROS and other inflammatory cytokines (e.g., TNF-α), the interaction between NF-κB and IκB is disturbed by the activation of certain enzymes known as IκB kinases (IKKs), which can phosphorylate and induce the degradation of IκB [79].

On the other hand, Nrf2 is the major antioxidant transcription factor that fights against oxidative stress by increasing the transcription of antioxidant enzymes, including HO-1, SOD, and CAT, by acting as the glutamate–cysteine ligase (GCL) which is needed for GSH synthesis [80]. As with NF-Kβ, Nrf2 nuclear translocation is regulated by a cytoplasmic protein known as the Kelch-like ECH-associated protein (keap1), which normally binds to Nrf2 and stimulates its ubiquitination and proteasome degradation [80]. ROS and many other flavonoids and drugs can activate the transcriptional potential of Nrf2 by either stimulating its transcription or disturbing the Nrf2–keap1 interaction [81,82,83]. Interestingly, the levels and activities of Nrf2 are significantly reduced, whereas the nuclear translocation of NF-κB is significantly reduced in the livers of rats with NAFLD and when associated with hepatic steatosis, inflammation, oxidative damage, and fibrosis [84,85,86,87,88,89]. However, the overexpression or pharmacological activation of Nrf2, or the deletion and suppression of NF-κB, alleviates hepatic steatosis and prevents the progression of NAFLD to NASH [84,85].

The important roles of Nrf2 and NF-κB in the development of NASH have also been confirmed in this study, where 16 weeks of feeding with an HFD resulted in similar effects in the livers of rats. On the contrary, treatment with ESAG was not only able to increase the mRNA and nuclear translocation of Nrf2, but it also reduced the nuclear translocation (activation) of NF-κB in the livers of HFD-fed rats. Such effects were observed in a dose-dependent manner, and similar results were also seen in the livers of control rats that received the highest dose of ESGA (200 mg/kg). This may explain why the livers of control and HFD-fed rats showed reductions in the levels of MDA, TNF-α, and IL-6 that were concomitant with higher levels of antioxidants such as GSH, SOD, CAT, and HO-1. Therefore, we suggest that ESGA protects against NAFLD through its antioxidant and anti-inflammatory effects, mediated by the activation of Nrf2 and suppression of NF-κB. Although these data are the first to show a possible regulatory effect of ESGA on the activities of Nrf2 and NF-κB, and despite the lack of such research in the literature, these findings are supported by previous studies using tomato extract. In this context, tomato powder reduced the expression of NF-κB and stimulated the Nrf2/antioxidant axis in healthy aged rats [90]. In addition, tomato phytochemical and electrophiles, including lycopene, β- and γ-carotenes, 16 byproducts of carotenoid oxidation, and 18 unknown compounds, were identified to stimulate the expression of Nrf2 in vivo in *C. elegans* [91]. Additionally, tomato paste reduced the expression of NF-κB in cultured cancerous prostate cells [92].

However, data have shown negative crosstalk between Nrf2 and NF-κB [93]. On the other hand, Nrf2 can alleviate inflammation by suppressing the activity and nuclear translocation of NF-κB by augmenting and preventing the phosphorylation of IκB via the downregulation and inhibition of IKK. A deficiency in Nrf2 induced NF-κB activation by increasing the phosphorylation and degradation of IκB [94]. Additionally, keap1 can inhibit NF-κB by targeting IKKβ [95]. In addition, Nrf2 can indirectly suppress NF-κB by stimulating the expression of HO-1, which stimulates the production of carbon monoxide and bilirubin, well-known suppressors of NF-κB nuclear translocation [93,96,97]. Supporting this, the activity but not the mRNA levels of NF-κB, as well as the levels of IL-1β, TNF-α, and IL-6, were increased in Nrf2-deficient astrocytes [98]. In addition, mice that were deficient in Nrf2 and sustained a head injury showed higher NF-κB activity [99]. Additionally, the overexpression of HO-1 reduced the expression of NF-κB, as well as the levels of IL-1β and TNF α in monocytes [96]. On the other hand, anti-inflammatory drugs stimulate Nrf2 activity by interfering with the nuclear translocation of NF-κB [93,100,101]. NF-κB p65 can directly inhibit Nrf2 at the transcriptional level by competing with the CH1-KIX domain of the transcriptional co-activator CBP [93,102].

Additionally, data from our study suggest that the activation of Nrf2 could be the major mechanism by which ESGA may act to inhibit hepatic inflammation and oxidative stress and is the major mechanism by which the nuclear transactivation of NF-κB is inhibited. Accordingly, treatment with ESGA not only stimulated the nuclear transactivation of Nrf2 but also increased its mRNA levels and reduced the level of keap1 in the livers of both control and HFD-fed rats. These effects were also associated with no significant alterations in the mRNA level of NF-κB but with a significant reduction in the hepatic activity of IKK in both treated groups (i.e., control and HFD-fed rats). Based on these data, we are confident that ESGA stimulates Nrf2 by increasing its transcriptional activity and reducing the level of keap1. In addition, it seems reasonable that ESGA suppresses the NF-κB/inflammatory axis through the Nrf2-mediated suppression of IKK and possibly by increasing the content of HO-1, as reported above. This could create a vicious cycle that exaggerates the activation of Nrf2 and inhibition of NF-κB, which ultimately prevents oxidative and inflammatory damage to the hepatocytes and alleviates hepatic steatosis. Importantly, such activation of Nrf2 could also explain the hypolipidemic potential of ESGA. Indeed, Nrf2, as it is also an antioxidant and anti-inflammatory agent, can suppress hepatic lipogenesis and steatosis and stimulate FA oxidation by improving mitochondrial biogenesis, inhibiting SREBP1, and upregulating PPARα. This may explain our previously discussed results concerning the expression of SREBP1 and PPARα in the livers of control and HFD-fed rats post-treatment with ESGA. However, the precise determination of the upstream regulator of either Nrf2 or NF-κB requires further, advanced experiments and will be considered in future studies using transgenic animals.

## 5. Conclusions

The data from this study are unique and the first to demonstrate a hepatic protective effect of ESGA against NAFLD in rats. We have shown that this newly discovered molecule has an exceptional ability to inhibit hepatic steatosis by suppressing oxidative stress and inflammation and by acting on the SREBP1, PPARα, Nrf2, and NF-κB signaling pathways. If confirmed in future clinical studies, this novel drug could act locally on the liver without modulating glucose and insulin levels and may be an excellent therapy option for other liver disorders.

## 6. Study Limitations

Despite our data, this study still has some limitations. One limitation is the inability to explain the absence of changes in the body and fat weight of HFD-fed rats despite the observed effect of this drug on the hepatic markers of -oxidation (PPARα β) and a decrease in the SREBP1/ACC pathway. Even though this could be explained by the lack of effect of ESGA on peripheral insulin sensitivity, the inhibitory effect of ESGA on hepatic SREBP1 signaling is explained by attenuating IR, ROS, and inflammation. Therefore, further studies investigating key molecular targets of adipogenesis in brown and white adipose tissues, such as PPARγ, which is normally expressed in the white and brown adipose tissue and is a major lipogenic gene, should be conducted to explain these observations. In addition, this study lacks the stability and bioinformatics of ESGA, which should be another target in future studies. In addition, the data presented here are still observational. Therefore, more advanced studies using human cells or mice deficient in Nrf2 and PPARα will help the field to confirm the precise mechanism of action of ESGA to fight NAFLD.

## Figures and Tables

**Table 1:** Primers used in the q-PCR reaction.

Target	Primer Sequence 5′→3′	Accession No.	Base Pair Length
PPARα	F:GAAGTCAAAGCCGACCCAAT R: AGGGTTCTTCCTTCGCACAC	NM_019142	116
CPT1	F:TCCGAGGCAGGAGCCCCATC R: TCTCGGTCCAGTTTGCGGCG	NM_013200.1	124
SREBP1c	F: GCTCACAAAAGCAAATCACT R: GCGTTTCTACCACTTCAGG	NM_001276707.1	191
ACC-1	F:TGAGGAGGACCGCATTTATC R: AAGCTTCCTTCGTGACCAGA	NM_022193.1	221
Nrf2	F:AAAATCATTAACCTCCCTGTTGAT R: CGGCGACTTTATTCTTACCTCTC	NM_031789	118
NF-κB	F:GTGCAGAAAGAAGACATTGAGGTG R:AGGCTAGGGTCAGCGTATGG	XM_342346.4	176
β-actin	F: CGAGTACAACCTTCTTGCAGC R: CCTTCTGACCCATACCCACC	NM_031144.3	209

**Table 2:** The effects of esculeogenin A (ESGA) on the final body weights, fat pad weights, and adipogenic markers in all experimental groups.

Parameter	Control	ESGA (200 mg/kg)	HFD	HFD + ESGA (50 mg/kg)	HFD + ESGA (100 mg/kg)	HFD + ESGA (200 mg/kg)
Final body weight (g)	506.2 ± 43.2	519.4 ± 55.9	634.5 ± 58.2 ^ab^	653.4 ± 61.9 ^ab^	638.7 ± 52.3 ^ab^	659.2 ± 54.7 ^ab^
Liver weight (g)	15.8 ± 1.3	16.4 ± 1.6	24.7 ± 1.9 ^ab^	19.8 ± 1.8 ^abc^	17.0 ± 1.4 ^abcd^	15.8 ± 1.1 ^bcde^
Liver/body weight %	3.04 ± 0.32	3.16 ± 1.6	3.93 ± 0.57 ^ab^	2.95 ± 0.11 ^bc^	2.68 ± 0.7 ^abcd^	2.34 ± 0.31 ^abcde^
Subcutaneous fat (g)	5.6 ± 0.46	5.8 ± 0.45	9.3 ± 0.89 ^ab^	8.7 ± 0.93 ^ab^	8.9 ± 0.85 ^ab^	9.2 ± 1.3 ^ab^
Epididymal fat (g)	7.2 ± 1.3	7.5 ± 1.1	13.4 ± 1.3 ^ab^	12.9 ± 1.4 ^ab^	14.2 ± 1.8 ^ab^	12.6 ± 1.2 ^ab^
Peritoneal fat (g)	4.7 ± 0.64	4.3 ± 0.48	8.45 ± 0.74 ^ab^	9.2 ± 1.1 ^ab^	8.7 ± 0.95 ^ab^	9.0 ± 1.2 ^ab^
Serum adiponectin (µg/mL)	45.3 ± 6.7	41.7 ± 8.1	20.1 ± 2.1 ^ab^	25.5 ± 3.5 ^ab^	19.6 ± 3.7 ^ab^	23.7 ± 3.1 ^ab^
Serum leptin (ng/mL)	22.7 ± 2.8	25.6 ± 4.8	64.5 ± 5.3 ^ab^	61.2 ± 7.8 ^ab^	58.9 ± 6.9 ^ab^	63.9 ± 6.5 ^ab^
Serum IL-6 (pg/mL)	2.4 ± 0.54	2.7 ± 0.18	28.6 ± 3.1 ^ab^	33.1 ± 4.3 ^ab^	30.8 ± 2.7 ^ab^	31.2 ± 4.1 ^ab^
Serum TNF-α (pg/mL)	0.82 ± 0.13	0.73 ± 0.09	4.9 ± 0.53 ^ab^	5.1 ± 0.84 ^ab^	4.4 ± 0.72 ^ab^	5.3 ± 0.69 ^ab^
Serum FFAs (mmol/L)	3.8 ± 0.57	4.3 ± 0.52	16.8 ± 2.1 ^ab^	18.2 ± 1.9 ^ab^	15.8 ± 2.6 ^ab^	16.9 ± 1.6 ^ab^

Data were analyzed via one-way ANOVA and Tukey’s post hoc test and are presented as the mean ± standard deviation (SD) for *n* = 8 samples/group. The level of significance was considered to be *p* < 0.05. ^a^ Significantly different from the control rats fed a standard diet. ^b^ Significantly different from the ESGA-treated rats. ^c^ Significantly different from the high-fat diet (HFD)-fed rats. ^d^ Significantly different from the HFD + ESGA (50 mg/kg)-fed rats. ^e^ Significantly different from the HFD + ESGA (100 mg/kg)-fed rats. HFD: high-fat diet; FFAs: free fatty acids.

**Table 3:** The effect of esculeogenin A (ESGA) on glucose and insulin levels as well as on insulin resistance and markers of liver function in all experimental groups.

Parameter	Control	ESGA (200 mg/kg)	HFD	HFD + ESGA (50 mg/kg)	HFD + ESGA (100 mg/kg)	HFD + ESGA (200 mg/kg)
FPG (mg/dL)	114.2 ± 9.4	110.8 ± 12.8	183.4 ± 22.3 ^ab^	176.7 ± 16.5 ^ab^	171.3 ± 18.6 ^ab^	180.1 ± 18.3 ^ab^
FPI (mg/dL)	3.8 ± 0.53	4.2 ± 0.49	7.3 ± 0.82 ^ab^	8.1 ± 0.94 ^ab^	7.1 ± 0.82 ^ab^	7.5 ± 0.88 ^ab^
HOMA-IR	1.03 ± 0.1	1.16 ± 0.23	3.31 ± 0.43 ^ab^	3.52 ± 0.31 ^ab^	3.11 ± 0.52 ^ab^	3.44 ± 4.72 ^ab^
Serum ALT (U/L)	29.9 ± 1.7	22.4 ± 2.8	127.3 ± 11.6 ^ab^	98.3 ± 13.2 ^abc^	57.3 ± 6.4 ^abcd^	26.3 ± 2.3 ^bcde^
Serum AST (U/L)	41.3 ± 5.2	47.6 ± 5.8	153.2 ± 14.1 ^ab^	103.5 ± 15.7 ^ab^	72.5 ± 8.4 ^abcd^	45.8 ± 18.4 ^bcd^
Serum GTT (U/L)	25.8 ±2.3	23.7 ± 3.6	88.3 ± 8.3 ^ab^	68.4 ± 5.4 ^ab^	46.9 ± 6.3 ^abcd^	27.3 ± 3.4 ^bcde^

Data were analyzed via one-way ANOVA and Tukey’s post hoc test and are presented as the mean ± standard deviation (SD) for *n* = 8 samples/group. The level of significance was considered to be *p* < 0.05. ^a^ Significantly different from the control rats fed the standard diet. ^b^ Significantly different from the ESGA-treated rats. ^c^ Significantly different from the high-fat diet (HFD)-fed rats. ^d^ Significantly different from the HFD + ESGA (50 mg/kg)-fed rats. ^e^ Significantly different from the HFD + ESGA (100 mg/kg)-fed rats. HFD: high-fat diet; FPG: fasting plasma glucose; FPI: fasting plasma insulin; HOMA-IR: The Homeostasis Model Assessment of Insulin Resistance. ALT: alanine aminotransferase; AST: aspartate aminotransferase; and GTT: gamma-glutamyl transpeptidase.

**Table 4:** The effects of esculeogenin A (ESGA) on the serum and hepatic lipid profiles of all experimental groups of rats.

	Parameter	Control	ESGA (200 mg/kg)	HFD	HFD + ESGA (50 mg/kg)	HFD + ESGA (100 mg/kg)	HFD + ESGA (200 mg/kg)
Serum	TGs (mg/dL)	76.4 ± 5.8	59.8 ± 5.4	225 ± 19.4 ^ab^	176.4 ± 15.6 ^abc^	127.2 ± 11.9 ^abcd^	85.6 ± 7.4 ^abcde^
	CHOL (mg/dL)	109.5 ± 8.6	88.2 ± 6.8 ^ab^	296.7 ± 18.9 ^ab^	206.3 ± 17.8 ^abc^	156.4 ± 13.8 ^abcd^	114.2 ± 12.3 ^abcde^
	LDL-c (mg/dL)	56.4 ± 5.7	33.7 ± 4.3 ^ab^	164.5 ± 13.8 ^abc^	128.4 ± 10.8 ^abc^	93.2 ± 8.4 ^abcd^	67.8 ± 6.2 ^abcde^
Liver	TGs (ng/g tissue)	403.1 ± 41.5	311.3 ± 28.5 ^ab^	1242.1 ± 134.5 ^abc^	892.1 ± 93.5 ^abcd^	638.1 ± 54.3 ^abcd^	459 ± 51.4 ^abcde^
	CHOL (ng/g tissue)	119.2 ± 12.5	89.2 ± 58.7 ^ab^	434.2 ± 56.1 ^abc^	309.2 ± 33.1 ^abc^	212.2 ± 22.4 ^abcd^	153.4 ± 15.2 ^abcde^
Stool	TGs (ng/g)	3.18 ± 0.46	3.3 ± 3.71	6.83 ± 0.73 ^ab^	7.2 ± 0.92 ^ab^	6.54 ± 0.83 ^ab^	6.99 ± 0.73 ^ab^
	CHOL (ng/g)	2.95 ± 0.36	2.72 ± 0.41	4.83 ± 0.39 ^ab^	4.66 ± 0.53 ^ab^	4.51 ± 0.68 ^ab^	4.77 ± 0.41 ^ab^

Data were analyzed via one-way ANOVA and Tukey’s post hoc test and are presented as means ± standard deviation (SD) for *n* = 8 samples/group. The level of significance was considered to be *p* < 0.05. ^a^: Significantly different from the control rats fed the standard diet. ^b^: Significantly different from the ESGA-treated rats. ^c^ Significantly different from the high-fat diet (HFD)-fed rats. ^d^ Significantly different from the HFD + ESGA (50 mg/kg)-fed rats. ^e^ Significantly different from the HFD + ESGA (100 mg/kg)-fed rats. HFD: high-fat diet; TGs: triglycerides; and CHOL: cholesterol.

## Data Availability

The datasets used and analyzed during the current study are available from the corresponding author upon reasonable request.
